# Retail promotions and perceptions of R.J. Reynolds' novel dissolvable tobacco in a US test market

**DOI:** 10.1186/1477-7517-8-10

**Published:** 2011-05-15

**Authors:** Laura M Romito, M Kim Saxton, Lorinda L Coan, Arden G Christen

**Affiliations:** 1 Oral Biology Department, Indiana University School of Dentistry, Indianapolis, Indiana, USA; 2 Indiana University Kelley School of Business, Indianapolis, Indiana, USA; 3 Department of Periodontics & Allied Health, Indiana School of Dentistry, Indianapolis, Indiana, USA

## Abstract

**Background:**

With declining cigarette sales, tobacco manufacturers have been developing and marketing new smokeless products, such as R. J. Reynolds' dissolvable tobacco, Camel Sticks, Strips and Orbs. This study assessed the availability, price and point-of-purchase promotional strategies for Camel Dissolvables, and investigated consumer awareness, interest and perception of these products in the Indiana test market.

**Methods:**

An exploratory retail audit of point-of-purchase promotions was conducted in a random sample of retailers from 6 store categories (n = 81) in the test market area. Data included: store type, location, product placement, forms/flavors carried, price, types and locations of advertisements and promotions, and ad messages. An Awareness-Attitude-Usage (AAU) survey was used to gauge consumer awareness and knowledge of tobacco products including Camel Dissolvables. Respondents were shown promotional materials from a package onsert and perceptions and interest in the Camel Dissolvables were assessed. An Intended Target Survey (ITS) compared subjects' perceptions of ad targets for several non-tobacco products, as well as Camel Snus, Camel No. 9 and Camel Dissolvables. Respondents were asked to identify each ad's intended target category, perceived targetedness, and purchase intent.

**Results:**

The products were carried by 46% of stores, most frequently gas stations (100%) and convenience stores (75%). They were shelved near smokeless tobacco (70%), cigarettes (25%) or candy (5%). Prices ranged from $3.59 -$4.19 per package; most stores carried at least 1 promotional item. Ad messages included: "Dissolvable Tobacco" (60%). "Free Trial" (24%), "Special Price" (24%), "What's Your Style?" (22%). At 14% of stores, free trial packs of Camel Dissolvables were offered with another Camel purchase. Awareness was reported by 42% of respondents (n = 243), and trial by 3%. Consumer interest was very low, but younger respondents (< 40 years) were more familiar with Camel Dissolvables (60% vs. 45% for those > 40 years, p < .01). Males, as well as current and former smokers had higher rates of interest and trial; only 1% of never smokers reported trial. In the ITS, only for the 3 tobacco product ads, was perceived targetedness for smokers significantly higher than for non-smokers. Smokers and nonsmokers perceived that the ads targeted smokers.

**Conclusions:**

Current retail promotional strategies for Camel Sticks, Strips & Orbs appear to be targeting a select audience, primarily current smokers. Overall, consumer awareness, interest and trial were low.

## Background

Increased tobacco taxation, smoke-free workplace policies, and clean air laws have all contributed to the decline in the U.S. adult smoking rate [1,2]. In response to these and other societal changes which are eroding the consumer base for cigarettes, tobacco manufacturers have been aggressively developing and marketing smokeless tobacco (ST) products such as snus and new forms of moist snuff [3]. Sales of moist snuff and other tobacco products have increased, perhaps offsetting as much as 30% of the decline in cigarette sales [4]. In addition, cigarette companies have purchased the two largest U.S. smokeless tobacco manufacturers and now control a large portion of the ST market [5,6]. These developments have the potential for broad public health implications including increasing smokeless tobacco use among youth and increasing dual use of ST and cigarettes among smokers. The use of ST, especially the newer forms, is considered to be less toxic than cigarettes and has been advocated as a harm reduction strategy. However, this has raised public health concerns because this approach may promote experimentation and initiation of ST use, nicotine dependence, progression to smoking, and long-term, concomitant use of cigarettes and ST [7]. While the harms of dual tobacco use are currently being debated, one widely held concern is that if smokers can comfortably substitute ST in situations where smoking is not permitted, they may be less likely to make a quit attempt. This could hinder further reductions in smoking prevalence and tobacco cessation and negate any potential health benefit of using ST as a means of harm reduction [7].

The popularity of traditional smokeless tobacco has been limited because many smokers are displeased by the litter and unpleasantness of having to spit frequently. Consequently, in 2006, R.J. Reynolds (RJR) introduced Camel Snus, a "spitless" form of smokeless tobacco. While U.S. demand for snus keeps growing every year [8], some smokers have said they didn't care for these pouched products because they had to be removed from the mouth after use. To overcome this objection, RJR has developed tobacco which simply dissolves in the mouth so that users do not have to contend with odor, smoke, spit, or litter. These innovative products are branded as Camel Sticks, Camel Orbs and Camel Strips to denote their different forms: a toothpick-like stick, a lozenge and an edible strip [9,10]. Along with Portland, Oregon and Columbus, Ohio, Indianapolis, Indiana serves as a national test market for RJR's new dissolvable line. Among these areas, Indiana boasts the highest tobacco use and currently stands as the U.S. state with the second highest adult smoking rate (26.1%) [11].

RJR has stated that Camel dissolvable tobacco, which delivers 0.6 mg -3.1 mg nicotine per piece, is intended for current smokers who want the option to continue using tobacco when and where smoking is not permitted [12]. However, it is unknown to which specific subgroups these products will be marketed or for whom they will have unintended appeal. While RJR contends that the products are neither marketed to nor attractive to youth, the candy-like appearance of these products and their ability to be used discretely may make them appealing to children and adolescents, potentially increasing youth tobacco use and accidental poisonings [13-16]. It has been shown that tobacco promotions are more effective at attracting new users than existing users, particularly in the under-19 age group [17]. Moreover, the incidence of dual cigarette and smokeless tobacco use appears to be higher among adolescents. Patterns of snuff vs. cigarette use suggest that compared to younger males who are not daily dual users, those who use ST and smoke on a daily basis have higher levels of serum cotinine and greater nicotine dependence [7].

Since these new dissolvable tobacco products are in direct contact with oral tissues, it is important to know what impact their use will have on oral and systemic health. Smoking and traditional smokeless/spit tobacco are causal for many oral conditions including periodontal disease and oral cancer [18]. While not harmless, newer smokeless tobacco products, including Swedish snus, have been shown to have considerably lower levels of carcinogens which may greatly reduce their health risks to users [19,20]. Research on one dissolvable tobacco product found that it delivered significantly less toxicants than cigarettes [21]. If the public perceives that dissolvable tobacco products are less harmful than smoking, it may enhance the social acceptability of smokeless tobacco use.

In order to better understand the point-of- purchase marketing and promotions of RJR's Camel Dissolvables tobacco as well as public perceptions of the products and their advertisements, this exploratory study included both an audit of retail outlets in the Indiana test market, as well as pilot survey of central Indiana residents. The goals of the study were to 1) assess the availability, price and point of purchase promotional strategies for Camel Dissolvables tobacco and 2) determine the consumer awareness, interest and perception of these new products.

## Results

### Point of Purchase Retail Audit

The final sample of 81 retail stores included 23 gas stations (28.6% of the sample), 15 drug stores (18.6%), 14 convenience stores (17.1%), 13 grocery stores (15.8%), 9 liquor stores (11.4%), and 7 smoke shops (8.6%). Note that most types of stores were represented at 15% or more of the sample. However, not all audited cities had liquor stores and smoke shops in their downtown areas. As a result of their relatively lower prevalence, liquor stores and smoke shops totals have been combined in summary analyses.

### Availability of Camel Dissolvables and Location within Stores

As shown in Table 1, of the 81 retail locations audited, approximately 46% (N = 37) carried the Camel Dissolvables product line. The products were most frequently sold at gas stations (100%) and convenience stores (75%). Camel Dissolvables were usually placed behind the counter, and displayed in close proximity to other smokeless tobacco products; however, in some stores they were located near candy displays. In all of the stores that carried any of the new dissolvables, Camel Orbs were carried and available in both flavors ("fresh" and "mellow").

**Table 1:** Camel Orbs, Strips & Sticks Retail Point-of-Purchase Promotions

Characteristic	%
**Incidence of stores that carry Camel Dissolvables (sample n = 81)**:	46%
**Gas Stations (sample n = 23)**	100%
**Convenience Stores (sample n = 14)**	75%
**Liquor/Smoke Shops (sample n = 16)**	29%
**Drug Stores (sample n = 15)**	23%
**Grocery Stores (sample n = 13)**	9%
**Forms carried**:	
**All 3 Forms - Orbs, Sticks, and Strips**	84%
**Two Forms - a combination of the three**	5%
**One Form only - only Orbs, if only one form carried**	11%
**Flavors carried for Orbs**:	
**Both "Mellow" and "Fresh"**	95%
**Only one flavor of Orbs**	5%
**Location within store**:	
**Behind the counter**	95%
**In cashier area**	84%
**Away from the cashier**	16%
**Other Products in closest proximity**:	
**Smokeless Tobacco**	70%
**Cigarettes**	25%
**Candy**	5%
**List Price**:	
**< $3.99**	11%
**$3.99**	65%
**> $3.99**	24%
**Number of types of Promotions displayed in stores**:	
**No advertisements**	16%
**One advertisements**	30%
**Two types of advertisements**	30%
**Three types of advertisements**	16%
**Four or more types of advertisement**	8%
Location of promotions within store*:	
**Right next to product**	84%
**On the outside door**	38%
**On outside window**	32%
**Above register**	16%
**Hanging down from ceiling**	14%
Key Messages in advertisements*:	
**"Dissolvable Tobacco"**	60%
**"Free Trial"**	24%
**"Special Price"**	24%
**"What's your Style?"**	22%
**"Now Available"**	11%

*Doesn't add to 100% since multiple advertisements existed in each store

When retail salespersons were asked how long the new Camel dissolvables had been carried, 54% were unsure, 16% indicated it was approximately 3 months and the remaining 30% indicated it had been approximately 6 months. These responses suggest RJR does not have a strong personal sell strategy inside retail stores. Instead, the company may be relying on in-store and out-of-store advertisements to generate product demand.

### Price

In any individual store, all three forms of Camel dissolvables were identically priced. However, prices varied between $3.59 and $4.19 per unit package. Within this range, $3.99 was the most common price point (65%).

### Promotions

Promotional items were similar from store to store, and a wide variety of promotional display items were used including: point of purchase displays, hanging signs, window signs and shelf flags. Choice of promotional display was at the discretion of the retailer and most vendors (84%) carried at least one promotional item (Table 1). Within-store ads were typically located right next to the new products (84%). Advertising messages varied, and the most common was simply "Dissolvable Tobacco". In addition, 14% of the stores offered free trial packs of Camel Dissolvables. These trial packs were separate packages given out with another Camel purchase. [Additional File 1 depicts how Camel Dissolvables were displayed including the free trial packs; Additional File 2 illustrates use instructions from package onsert] Anecdotally, as a means of moving product off the shelves, some stores had started to give away an entire package of Camel Dissolvables to people who purchased a Camel product.

### Relationship between Smoking Prevalence and Camel Dissolvables Distribution

The percentage of retail locations carrying the test marketed product varied with central Indiana county smoking rates. Smoking prevalence was significantly correlated with the percentage of stores carrying Camel Dissolvables (r = 0.55, p < .001) [Additional File 3 and Additional File 4]. Although this finding suggests that counties with higher smoking rates have more stores that carry these new dissolvable tobacco products, this finding is correlational not causal. However, it is likely that a product targeted to existing smokers would be more easily found in geographical locations where more smokers are present.

### Survey Results

#### AAU Survey

Table 2 reports sample characteristics, rates of awareness and trial for Camel Dissolvables and likelihood of purchasing Camel Dissolvables after exposure to promotional materials from a color-printed package onsert. Awareness of Camel Dissolvables was reported by 42% of respondents (n = 243), and 3% had tried the new products. Interest in these new tobacco forms was very low with a mean likelihood of trial at 1.48, between definitely would not and probably would not. Only 7% of respondents indicated they probably or definitely would try Camel Dissolvables.

**Table 2:** AAU Results - Camel Dissolvables Awareness, Use and Interest by Subgroups

		Heard of Camel Dissolvables^1^		Tried Camel Dissolvables^2^		Likelihood of Trying Camel Dissolvables^3^	
				
Variable	% (*n*)	*% (95% CI)*	*p*	*% (95% CI)*	*P*	*Mean (95% CI)*	*p*
**Total**	100% (243)	42%		3%		1.48	
**Smoking Status**			***ns***		***< .01***		***< .001***
*Never*	67% (159)	38% (31-46%)		1% (0-2%)		1.24 (1.13-1.35)	
*In past, not now*	21% (59)	53% (39-68%)		10% (1-19%)		1.64 (1.34-1.94)	
*Smoke daily or some days*	12% (29)	48% (29-68%)		7% (0-17%)		2.55 (2.02-3.09)	
**Gender**			***ns***		***< .01***		***< .05***
*Male*	46% (112)	61% (45-78%)		7% (2-12%)		1.63 (1.43-1.84)	
*Female*	54% (131)	45% (35-55%)		0%		1.34 (1.20-1.48)	
**Age**			***< .01***		***Ns***		***ns***
*18-39 years old*	77% (187)	60% (49-71%)		4% (1-7%)		1.52 (1.38-1.67)	
*40 years and older*	23% (56)	28% (15-40%)		0%		1.30 (1.10-1.51)	
**Sample Source**			***ns***		***Ns***		***ns***
*Dental School Patients*	35% (84)	38% (27-48%)		1% (0-4%)		1.44 (1.23-1.65)	
*Dental School Students*	28% (69)	40% (28-52%)		0%		1.41 (1.18-1.63)	
*Business School Students*	37% (90)	49% (38-59%)		8% (2-14%)		1.56 (1.35-1.76)	
**Received Any Promotion**			***< .001***		***< .001***		***< .05***
*No*	69% (164)	21% (14-27%)		0		1.40 (1.25-1.54)	
*Yes*	31% (75)	92% (85-98%)		11% (4-18%)		1.67 (1.42-1.91)	
**See ad in Store**			***< .001***		***< .001***		***<.01***
*No*	75% (179)	25% (19-32%)		1% (0-2%)		1.39 (1.25-1.52)	
*Yes*	25% (59)	95% (89-100%)		12% (3-21%)		1.78 (1.49-2.07)	
**Received promotion at bar**			***< .001***		***< .001***		***< .01***
*No*	86% (205)	34% (27-40%)		1% (0-2%)		1.40 (1.28-1.53)	
*Yes*	14% (33)	100%		19% (4-33%)		1.97 (1.54-2.40)	
**Received promotion in mail**			***< .001***		***< .001***		***ns***
*No*	84% (201)	33% (27-40%)		2% (0-3%)		1.44 (1.31-1.57)	
*Yes*	16% (37)	94% (87-100%)		14% (2-26%)		1.70 (1.33-2.08)	
**Saw ad in Magazine**			***< .001***		***< .001***		
*No*	75% (179)	27% (20-33%)		2% (0-4%)		1.42 (1.28-1.56)	***ns***
*Yes*	25% (59)	93% (86-99%)		9% (1-16%)		1.68 (1.40-1.96)	

^1^Question asked was "*How familiar are you with ****dissolvable tobacco ****products including Camel Orbs, Camel Sticks and Camel Strips, etc.?" *5-point scale of Never to Know a lot about it. Recoded to 0/1 Never/Any familiarity. % reported is % any familiarity.

^2^Question asked was "*Tobacco companies have recently introduced spitless*, ***dissolvable tobacco ****products (currently sold as Camel Orbs, Camel Strips, Camel Strips, etc.). Have you ever tried any of these products?" *No-Yes. % reported is % yes, have tried.

^3^Respondents were shown color printed descriptions of the entire Camel Dissolvables product offering included as a package onsert (see Figure 1). Then, the question asked was "*If given the opportunity how likely would you be to try one of these ****dissolvable tobacco products****" *5-point scale where 1 = Definitely would not and 5 = Definitely would.

Younger respondents (< 40 years) were more familiar with Camel Dissolvables (60% vs. 45% for those ≥ 40 years, p < .01). Males, current smokers and former smokers had higher trial rates and higher interest in trying Camel Dissolvables: 7% of men had tried the products vs. 0% for women (p < .01). Male likelihood of trial was 1.63 vs. 1.34 (p < .05) for females; 10% of former smokers and 7% of current smokers had tried Camel Dissolvables while only 1% of non-smokers had tried them (p < .01). Current smokers were the most interested in trying at 2.55 vs. 1.62 for former smokers and 1.24 for non-smokers (p < .01). There were no significant differences in awareness, trial, or interest in trying Camel Dissolvables among other respondent subgroups.

In the AAU survey, respondents were also asked about their exposure to promotions for Camel Dissolvables in stores, by mail, at bars or in magazines. As shown in Table 2, those receiving any type of promotion were more familiar, more likely to have tried and more likely to try Camel Dissolvables. More importantly, promotions experienced at point of purchase or in-person in bars resulted in higher likelihood of trying Camel Dissolvables. Thus promotions, especially in-person, appear to promote trial of new smokeless forms of tobacco [22].

#### Intended Target Survey (ITS)

The results of the ITS suggest that people generally perceived that all 6 ads targeted existing category users since all means were greater than 4.0 (Table 3). The tobacco and ESPN ads were rated highest and were significantly higher than for TicTac or Crest with Scope (p < .001). In the total sample, perceived targetedness and purchase likelihood were highest for the Crest with Scope ad. Perceived targetedness was lowest for the ESPN ad and all three tobacco ads. Purchase likelihood was lowest for the tobacco ads.

**Table 3:** ITS Results - Intended Target, Felt Targetedness and Purchase Intent for Tobacco and Non-Tobacco Ads

Variables	N	TicTac Mean (95%CI)	ESPN Mean (95%CI)	Crest with Scope Mean (95%CI)	Camel Snus Mean (95%CI)	Camel Dissolvables Mean (95%CI)	Camel No. 9 Mean (95%CI)
**Total Sample**: differences across ads are significant at p < .001. Highest means have been bolded.							
Intended Target: Category Users^1^	49	4.31 *(3.91-4.71)*	**5.28 *(4.92-5.63)***	4.41 *(3.90-4.92)*	**5.41 *(4.94-5.88)***	**5.29 *(4.80-5.77)***	**5.33 *(4.88-5.77)***
Felt Targetedness^2^	65	3.56 *(3.33-3.79)*	2.32 *(2.03-2.60)*	**3.82 *(3.68-3.96)***	2.29 *(2.01-2.57)*	2.41 *(2.11-2.71)*	2.36 *(2.04-2.68)*
Likelihood of Purchase^3^	65	3.34 *(3.09-3.60)*	2.56 *(2.26-2.86)*	**3.64 *(3.42-3.85)***	1.48 *(1.26-1.71)*	1.44 *(1.24-1.64)*	1.41 *(1.23-1.59)*
							
**Smokers vs. Non-smokers**							
Intended Target: Category Users		p = ns	p = ns	p = ns	**p < .05**	p = ns	p = ns
*Smokers*	14	4.57 *(3.76-5.38)*	5.14 *(4.27-6.02)*	4.93 *(4.04-5.82)*	**6.21 ***(5.65-6.78)*	5.71 *(5.10-6.33)*	5.14 *(4.13-6.15)*
*Non-Smokers*	34	4.21 *(3.73-4.68)*	5.24 *(4.81-5.66)*	4.18 *(3.53-4.83)*	5.26 *(4.71-5.82)*	5.18 *(4.53-5.82)*	5.24 *(4.70-5.77)*
							
Felt Targetedness		p = ns	p = ns	p = ns	**p < .01**	**p < .001**	**p < .05**
*Smokers*	18	3.56 *(3.04-4.07)*	2.41 *(1.85-2.96)*	3.93 *(3.74-4.11)*	**3.03 ***(2.45-3.62)*	**3.19 ***(2.57-3.80)*	**2.94 ***(2.22-3.67)*
*Non-Smokers*	47	3.55 *(3.28-3.82)*	2.31 *(1.96-2.65)*	3.77 *(3.58-3.96)*	2.06 *(1.75-2.36)*	2.12 *(1.80-2.44)*	2.10 *(1.77-2.43)*
							
Likelihood of Purchase		p = ns	p = ns	p = ns	**p < .05**	**p < .05**	p = ns
*Smokers*	18	3.33 *(2.77-3.90)*	2.39 *(1.87-2.90)*	3.89 *(3.55-4.23)*	**1.94 ***(1.30-2.59)*	**1.83 ***(1.24-2.43)*	1.61 *(1.22-2.00)*
*Non-Smokers*	46	3.35 *(3.06-3.64)*	2.62 *(2.25-2.98)*	3.57 *(3.31-3.84)*	1.32 *(1.13-1.50)*	1.28 *(1.11-1.45)*	1.34 *(1.14-1.54)*

^1^Question asked was *" When they create ads, advertisers generally have a particular audience they are trying to talk to with their ad. Who do you think __ad is aimed at?" *Scale was 7-point semantic differential anchored by 1 = People who don't use ___category and 7 = people who already use __category

^2^Felt Targetedness was measured by 3 items asked on a 5-point Likert scale: *"I feel this ad was intended for people like me. I believe this ad was targeted to people like me. This as was meant to appeal to people like me." *Since Cronbach α = 0.90, all three items were averaged for the Felt Targetedness scale.

^3^Question asked was *"Based on this ad, how likely are you to purchase ___?" *Scale was 5 points: Very unlikely, Unlikely Undecided, Likely and Very Likely

There were no significant differences between smokers and non-smokers in ratings for the TicTac, ESPN or Crest with Scope ads for intended target in category users, perceived targetedness and purchase likelihood. However, for all three tobacco product ads, perceived targetedness for smokers was significantly higher than for non-smokers. In addition, among smokers, purchase likelihood was also higher for Camel Snus and Camel Dissolvables. Intended target as category user was also higher among smokers for the Camel Snus ad. Thus, smokers perceived that they are targeted by all three tobacco ads. Non-smokers also perceived that smokers are targeted by ads for these new tobacco forms.

## Discussion

For decades, cigarette companies have spent millions of dollars per annum on ST research, consumer profiling, product development, and marketing [23]. In the face of greater smoking restrictions and declining cigarette sales, they have developed new cigarette-branded smokeless, spitless products in an effort to satisfy consumer preferences whilst attempting to expand their consumer base [23]. RJR's latest contribution to the smokeless tobacco market is a new line of dissolvable tobacco products: Camel Orbs, Strips and Sticks. As with other forms of tobacco which are usually purchased on an as-needed basis, this study found that the Camel Dissolvable tobacco products were most commonly sold at so-called "on-the-go" retailers - gas stations and convenience stores. These products were often co-located with Camel cigarettes and other forms of tobacco, such as Camel Snus, thereby emphasizing recognition of their popular brand.

Of particular concern was the co-location of Camel Dissolvables in 5% of stores with candy. Consumer advocacy groups and governmental agencies have expressed alarm that these new forms of smokeless tobacco may be confused with mints and candies [9,24,25]. The candy-like appearance of Camel Dissolvables and their ability to be used discretely may make them appealing to children and adolescents, potentially increasing tobacco use and/or accidental poisoning in youth [16,25,26]. Due to these concerns, and their expanded authority over tobacco regulation, the U.S. Food and Drug Administration (FDA) called for RJR to provide detailed reports regarding its knowledge of youth perceptions, use, and misuse of Camel Dissolvables [24]. While the products were located behind the retail counter and sold in child-resistant packaging, their appearance and retail proximity to candy may enhance their risks to youth. In addition, point-of-purchase tobacco promotions have been shown to increase acceptance of tobacco among youth and encourage tobacco use [27,28].

Nearly two-thirds of retailers offered the products at the manufacturer's suggested retail price of $3.99; 11% of stores discounted below that price point and another 24% charged a premium for these products. Further, 24% of stores advertised a "special price", typically equated with reduced prices. Similar to the initial marketing of snus [29], retailers reported that Camel Dissolvables did not move well without incentives such as discounts or coupons for a free container. As a result, 14% of stores were running a "free trial" promotion. In some cases, retail salespeople had been instructed to simply give the product away to get rid of it. However, while retailers generally described demand for the products as low, history has shown that such a response is not unusual following the introduction of a new tobacco product; with consumer feedback, further product improvements and effective marketing campaigns, product popularity and subsequently, sales, can increase over time [23]. In addition, the use of discount pricing strategies may further enhance trial and ultimately, longer-term use.

The vast majority of stores (84%) displayed ads for the products, typically, adjacent to the product itself. One fourth of the retailers showcased more extensive in-store advertisements - three or more different ads with secondary locations in store windows or doors. The majority of ad messages simply announced the new product's availability ("Dissolvable Tobacco"), while others emphasized price ("Special Price") or consumer characteristics ("What's Your Style?"). While RJR appears to be promotionally supporting its newest smokeless product offering, this support is not typical of mass marketed new product introductions. Rather, promotions suggest a strategy targeted toward a select consumer audience, such as current smokers. In essence, RJR appears to be trying to capture more tobacco use instances per smoker or a dual use strategy of both smoking and smokeless tobacco.

Currently, there is no consensus on the health impact of dual tobacco use patterns in existing smokers. Furthermore, the issue is complicated by the fact that varying definitions of dual use in the scientific literature have generated different prevalence estimates and risk profiles [30]. Nonetheless, it has been suggested that dual tobacco use may discourage tobacco cessation, increase nicotine levels, and exposure to tobacco toxicants [7]. However, a recently published review of the scientific literature by tobacco industry researchers found no "unique health risks associated with dual use of smokeless tobacco products and cigarettes, which are not anticipated or observed from cigarette smoking alone" [31]. From studies of tobacco use trajectory data, these authors also concluded that compared to those who only smoke cigarettes, dual users are more likely to quit smoking [31]. However, their interpretation of the data has been questioned by others who contend that promotional strategies which support dual use encourage continued tobacco use in individuals who, in response to expanding smoke-free environments, would otherwise have quit [32]. Other studies concur. For example, a national survey of dual tobacco users found that most used ST in places where smoking was not permitted and most did not believe ST was a useful cessation aid. In addition, compared to exclusive cigarette smokers, fewer dual users reported planning to quit in the next 6 months and nearly half did not plan to quit smoking at all [33]. An investigation of the changes in tobacco use patterns over time among a cohort of US Air Force personnel found that of the smokers who initiated ST use following basic training, 87% became dual users, a result which the researchers classified as "harm escalation". Military personnel who quit smoking and/or quit dual use to become exclusive ST users were classified in the "harm reduction" group and they represented only 13.2% of the study population [34].

Inferences about the harm reduction potential of Camel Dissolvables may be made from how consumers perceive them. If the products' use becomes widespread, several outcomes are possible. Consumption by new users or former tobacco users may increase the burden of nicotine dependence in the population. Use by current smokers may result in cessation, switching to exclusively using Dissolvable tobacco or dual use. It appears that such products are not employed as a tool for cessation, and switching from smoking to ST use could be beneficial or harmful depending upon who is using the products, how they are used as well as the levels of nicotine and toxicants in the products, and product regulatory controls. At a minimum, dual use appears to be a means to maintain tobacco dependence. In the present study current and former smokers appear most interested in the products, so dual use and perhaps even relapse are potential outcomes. While we found that the rates of interest and trial of Camel Dissolvables were low, the aforementioned studies suggest that if smokers' awareness, interest, and satisfaction with these products grow, more smokers will be engaged in dual tobacco use which may negate any health benefits from using the lower toxicity Dissolvables. These individuals may be more likely to remain tobacco users, and public health efforts toward tobacco cessation may be undermined.

Interestingly, the AAU survey found higher levels of awareness and trial for Camel Dissolvables than Snus at approximately the same point in test marketing [22]. This may be due to sample demographics. University campuses have been reported to hold events where the products were promoted with free samples, coupons, etc. While product awareness, trial and interest were all quite low, they were highest among young adults and male smokers. These results are consistent with previous studies which found that current or previous male smokers are more likely to try new forms of smokeless tobacco [3,7,35,36]. Promotions are also linked to familiarity, trial and likelihood of trying Camel Dissolvables. In fact, all of the consumers who had tried Camel Dissolvables had received some type of promotion. Trial rates for those who had received any promotion were almost four times higher than the total sample (11% vs. 3%). The ITS further reinforced these findings; respondents not only believed these ads are targeted to smokers, but smokers themselves feel more targeted and are more likely to purchase these new smokeless tobacco products.

This exploratory study has several limitations. The primary retail point-of-purchase marketing strategies for RJR's Camel Dissolvable tobacco products were evaluated in only one U.S. test market. Therefore, these results may not be fully representative to the universe of tobacco retailers. In addition, the field audit did not include a detailed comparative analysis of the new products with popular cigarette products. However, our findings are consistent with the stated marketing plans of RJR and provide a snapshot of the ongoing test marketing of Camel Orbs, Sticks and Strips. Further research is needed to monitor marketing strategies and sales outcomes of these products over time. Study findings also suggest that promotions, especially those aimed at trial (i.e. in-store ads and in-bar promotions) play a major role in creating awareness and product trial. In-store and bar promotions are also consistent with a younger smoker target for Camel Dissolvables. Although these results do provide some insight into the marketing of Camel Dissolvables, they are exploratory in nature and are limited by the relatively small sample size as well as the sample selection and demographics.

While the primary audience for the point-of-purchase retail advertising and promotion of the new dissolvable products appears to be existing smokers, these promotions may increase visibility of the products to youth. The ads, candy-like appearance of the Camel Dissolvables, and their ability to be used discretely may encourage new young users. In addition, as Camel Dissolvables are promoted as a means to use tobacco where smoking is not permitted or acceptable, they may hinder quit attempts in existing smokers and promote dual use of both cigarettes and smokeless tobacco. While the long-term public health consequences of dual tobacco use have not yet been established, public health and tobacco control researchers have advocated that in order to further reduce population harms from tobacco use, ST marketing activities aimed at new users or promoting dual use, including dissemination of free samples, providing consumers' instruction in product use, using youth-appealing messages, new flavorings and low nicotine levels, should be restricted [23]. These activities are now subject to regulation as a result of the Family Smoking Prevention and Tobacco Control Act, enacted in 2009, which gave the FDA broad authority over the manufacture, marketing, distribution, sale, and importation of tobacco products. One major area of focus for the FDA is in evaluating products such as the Dissolvables, which are purported to reduce harm or the risk of tobacco-related diseases as compared with other commercially marketed tobacco products. To do so, the FDA must have sufficient data to understand the public health effects of such products as well as their appeal to youth [37]. Information on the marketing and promotional strategies of new smokeless tobacco products, such as Camel Orbs, Strips and Sticks, and the impact of these products on public perceptions and tobacco use behaviours may better inform regulators and health professionals' policy and practice decisions in order to reduce future tobacco-related morbidity and mortality.

## Conclusions

Current retail promotional strategies for RJR's Camel Orbs, Strips and Sticks suggest a more selective, rather than intensive distribution, targeted toward existing smokers. Surveys indicated that both smokers and non-smokers perceived Camel Dissolvables promotions as targeting smokers. However, consumer awareness of Camel Dissolvables during test marketing was very low; males and current and former smokers had greater awareness, interest and trial of the products.

## Methods

The study was conducted in two phases. In the initial phase, an audit of tobacco retailers' point of purchase advertising and promotions was performed; consumer surveys constituted the second phase. The field audit consisted of a random sampling of retailers representing six different store types (gas stations, convenience and grocery stores, liquor stores, drug stores and tobacco shops) in the eight counties surrounding and including Indianapolis. The field audit took place approximately one year after the start of the test market from December 15, 2009 to January 15, 2010. In each county, the most densely populated cities were identified to serve as the field audit locations. A field audit protocol was developed to ensure researcher calibration, systematic and parallel data collection across all audit localities. Each researcher was instructed to randomly select two stores from each of the six retail categories in each audit locality. At each store audited, researchers recorded the following data elements: store type and location, product placement within the store, product forms and flavors carried, price, types of advertisements and promotions (posters, point-of-purchase displays, shelf flags, hanging signs, window signs, and samples/coupons), location of advertisements (window, door, above register, ceiling, and next to product), and specific ad messages. Researchers also asked store employees about the length of time the products had been available at their store and their perception of product sales/popularity. Where permissible, researchers took digital photographs of product displays and advertisements. The final sample included 81 stores representing six different store types. The data was then entered into an Excel spreadsheet for cataloguing, coding and content analysis. Analysis included frequency counts and percentages. In addition, adult smoking prevalence by county from annual CDC BRFSS was compared to the percentage of stores in each county carrying the products. Given the size of the final sample, all percentages were rounded to whole numbers.

**Figure 1:**
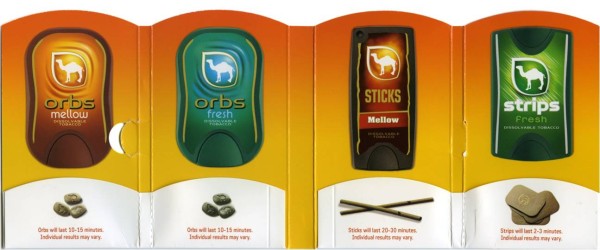
**Camel Dissolvables Promotional Package Onsert**.

In the second phase, two surveys were used to better understand consumer awareness, interest and perceptions of the Camel Dissolvables product line. A 17-item Awareness-Attitude-Usage (AAU) and an Intended Target Survey (ITS) were developed using items from previously validated and published instruments [17,22,38-41]. In addition, specific questions about Camel Dissolvables tobacco were incorporated. After gauging consumers' awareness and knowledge of tobacco products including the Camel Dissolvables, respondents were shown promotional materials from a color-printed package onsert (Figure 1). Subsequently, respondent perceptions and interest in the Camel Dissolvables were assessed. The AAU survey was pretested with 25 Indiana University Purdue University Indianapolis (IUPUI) student volunteers and revised based on volunteer feedback. The AAU survey was then administered to a convenience sample of 243 consumers including 159 IUPUI students and 84 patients of the Indiana University School of Dentistry (IUSD). All participants completed the entire survey. The ITS was administered to a separate convenience sample of 65 IUPUI undergraduates. All participants completed the entire survey. The ITS compared subjects' perceptions of ad targets for several non-tobacco products, as well as other newer Camel products such as Camel Snus and Camel No. 9 cigarettes. Subjects were shown six actual print ads for the following: Tic Tac, ESPN, Crest with Scope, Camel Snus, Camel Dissolvables and Camel No. 9. Respondents were asked to identify each ads' intended target category (i.e. users vs. non-users), perceived targetedness (does the ad target them), and purchase intent. All data were summarized via descriptive statistics including counts, frequencies and means. Difference between subgroups were tested via ANOVA with statistical significance set at p ≤ .05.

## Competing interests

The authors declare that they have no competing interests.

## Authors' contributions

LR designed the study, performed and supervised study procedures, and wrote the manuscript. MKS performed and supervised study procedures, developed survey instruments, conducted data analyses, and contributed to the writing and editing of the manuscript. LLC supervised study procedures, contributed to the manuscript. AC served as consultant to study methods and procedures, and edited the manuscript.

## Supplementary Material

Additional file 1 **Retail Displays of Camel Dissolvables and Free Trial Pack**. Click here for file

Additional file 2 **Camel Dissolvable Tobacco Use Instructions from Package Onsert**. Click here for file

Additional file 3 **County Smoking Rates compared to Camel Dissolvables Distribution (graph)**. Click here for file

Additional file 4 **County Smoking Rates compared to Camel Dissolvables Distribution (table)**. Click here for file
