# Supplementary figures and images for: Retail promotions and perceptions of R.J. Reynolds' novel dissolvable tobacco in a US test market
Source: Harm Reduct J. 2011 May 15;8:10. doi: 10.1186/1477-7517-8-10 (PMC3123190; doi:10.1186/1477-7517-8-10)

Additional File 1: Retail Displays of Camel Dissolvables and Free Trial Pack


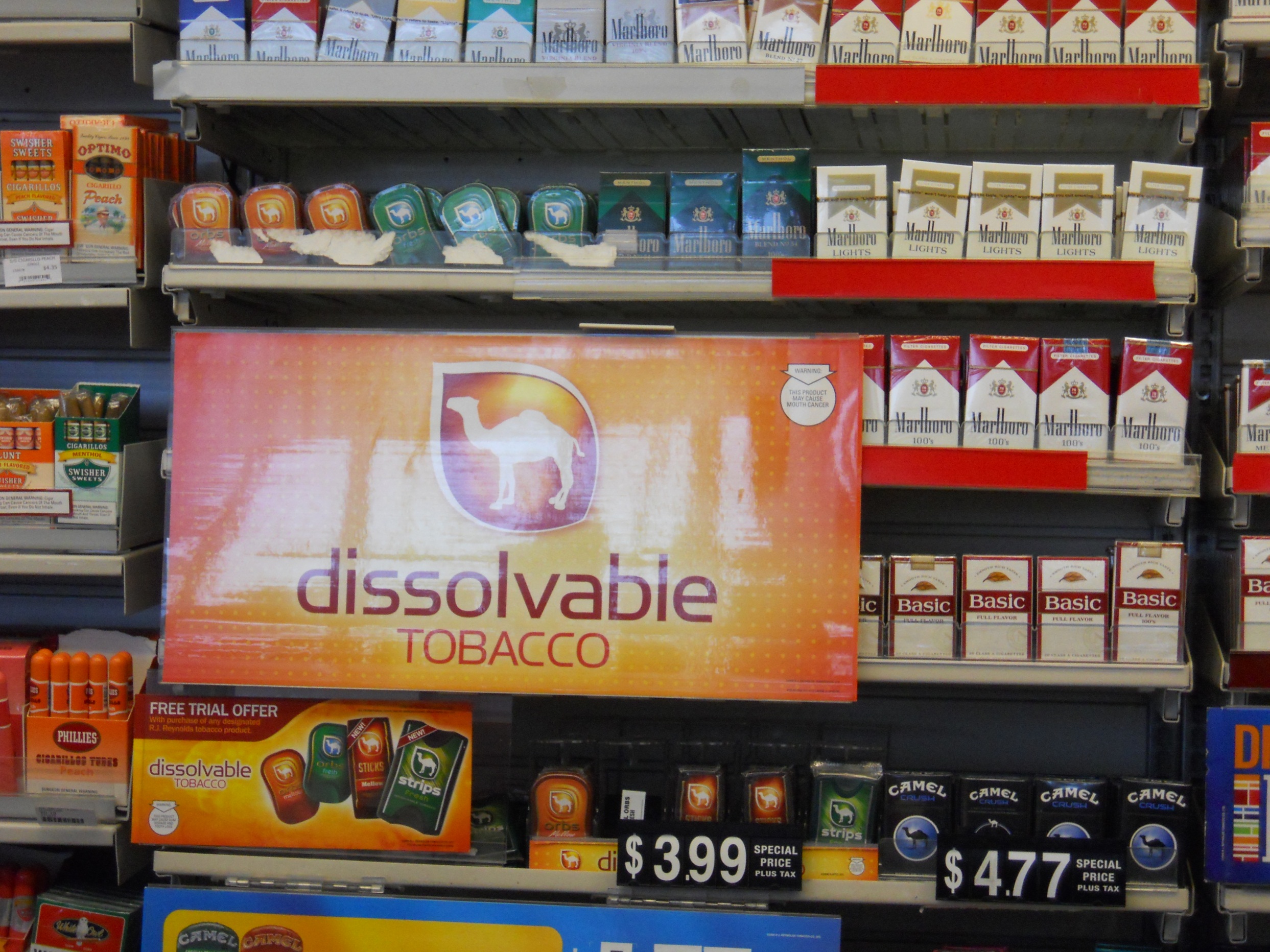


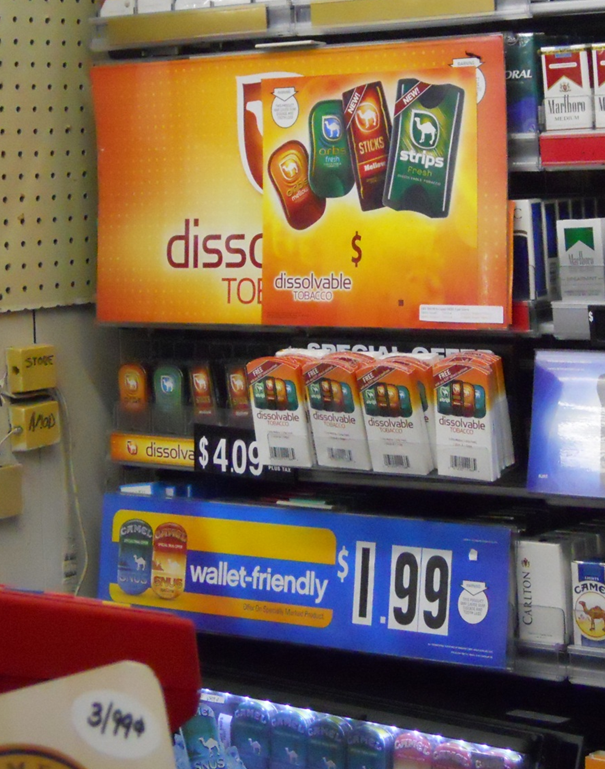

Supplement: Additional file 1 — Retail Displays of Camel Dissolvables and Free Trial Pack. [file 1477-7517-8-10-S1.DOC]

Additional File 2: Camel Dissolvable Tobacco Use Instructions from Package Onsert


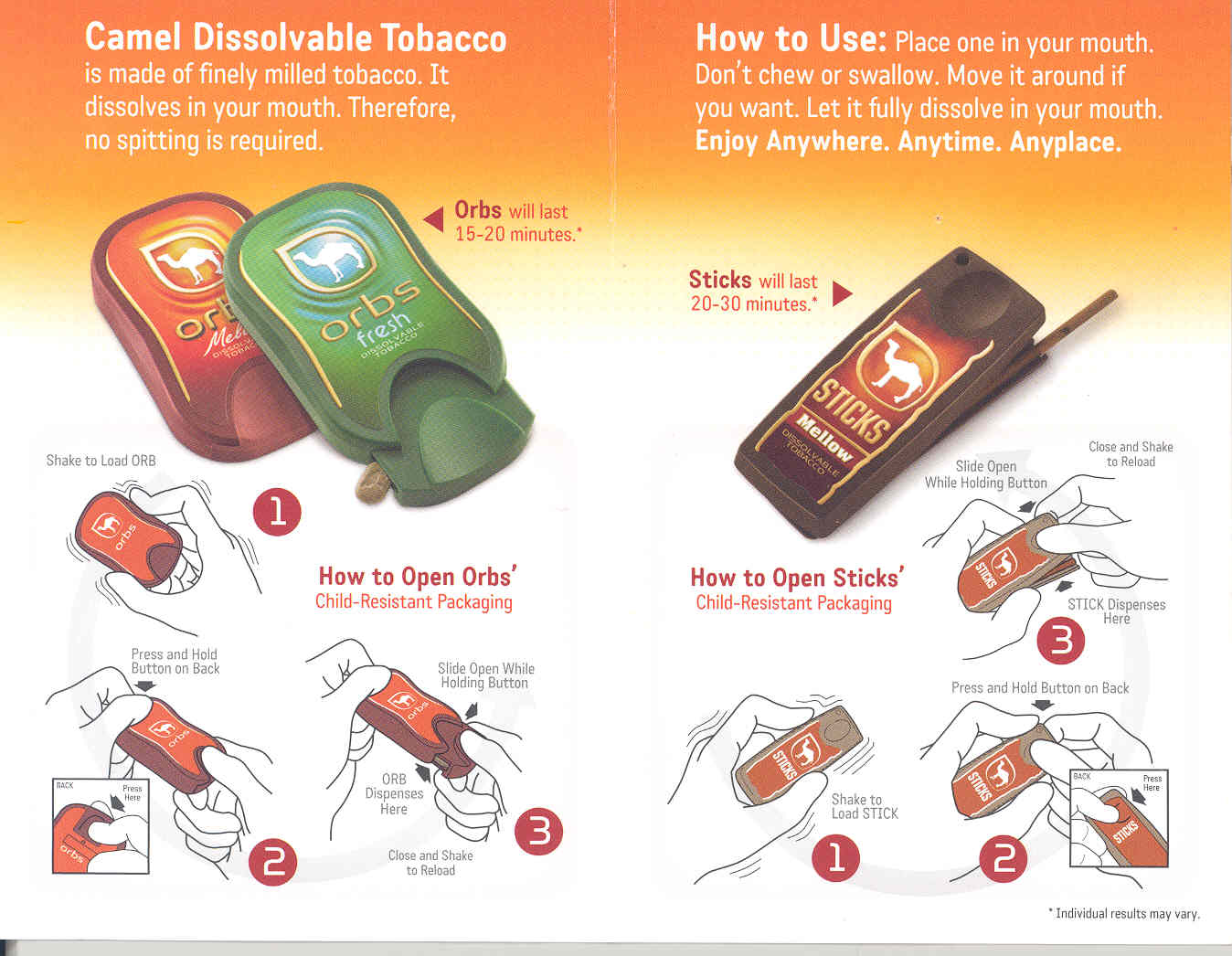

Supplement: Additional file 2 — Camel Dissolvable Tobacco Use Instructions from Package Onsert. [file 1477-7517-8-10-S2.DOC]

Additional File 3: County Smoking Rates compared to Camel Dissolvables Distribution


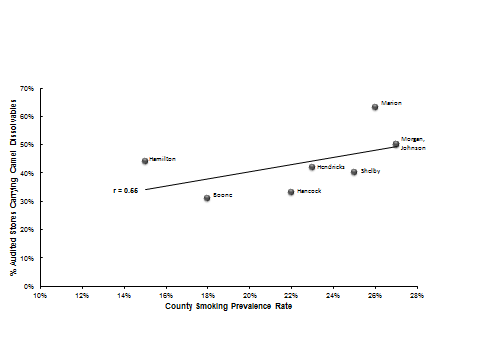

Supplement: Additional file 3 — County Smoking Rates compared to Camel Dissolvables Distribution (graph). [file 1477-7517-8-10-S3.DOC]
