# Supplementary material for: Retail promotions and perceptions of R.J. Reynolds' novel dissolvable tobacco in a US test market
Source: Harm Reduct J. 2011 May 15;8:10. doi: 10.1186/1477-7517-8-10 (PMC3123190; doi:10.1186/1477-7517-8-10)
Supplement: Additional file 4 — County Smoking Rates compared to Camel Dissolvables Distribution (table). [file 1477-7517-8-10-S4.DOC]

Additional File 4: County Smoking Rates compared to Camel Dissolvables Distribution

| **Indiana County** | **Adult smoking rate: 2002-2008 BRFSS (est.)** | **Number of adult smokers (est.)** | **% of Stores that carried Camel Dissolvables** |
| --- | --- | --- | --- |
| Boone | 18% | 5,949 | 31% |
| Hamilton | 15% | 18,972 | 44% |
| Hancock | 22% | 8,953 | 33% |
| Hendricks | 23% | 17,233 | 42% |
| Johnson | 27% | 22,647 | 50% |
| Marion | 26% | 166,063 | 63% |
| Morgan | 27% | 13,105 | 50% |
| Shelby | 25% | 3,749 | 40% |
| **Indiana Total** | **26%** | **1,171,583** | 46% |
| SOURCES: Indiana County Health Rankings Report, 2009 BRFSS & study field audit | | | |
